# Supplementary material for: Incidence of SARS-CoV-2 Infection and Related Mortality by Education Level during Three Phases of the 2020 Pandemic: A Population-Based Cohort Study in Rome
Source: J Clin Med. 2022 Feb 7;11(3):877. doi: 10.3390/jcm11030877 (PMC8836834; doi:10.3390/jcm11030877)
Supplement: Supplementary file 1 [file jcm-11-00877-s001.zip › jcm-1554469-supplementary.pdf]

SUPPLEMENTARY MATERIAL

SUMMARY

Table S1: SARS-CoV-2 infections by educational level, sex, and age. Crude cumulative incidence (CCI) with 95% CI and $\chi 2$ p-value for comparison. Rome, 35+ years old, 1-03-2020 to 31-12-2020. 2

Table S2. Univariate and multivariable log-binomial regression model. RR, 95% CI and p-value. 4

Table S3: Deaths within 30 days of infection by educational level, sex, and age. Crude mortality rate (CMR) with 95% CI and and $\chi 2$ p-value. Rome, 35+ years old, 1-03-2020 to 31-12-2020. 6

Table S4: Univariate and multivariable Cox regression model. HR, 95% CI and p-value 8

Table S1: SARS-CoV-2 infections by educational level, sex, and age. Crude cumulative incidence (CCI) with 95% CI and χ2 p-value for comparison. Rome, 35+ years old, 1-03-2020 to 31-12-2020.

				**ENTIRE PERIOD**					
				**SARS-CoV-2 INFECTION**					
	**No**		**Yes**		**All**				
	**N**	**%**	**N**	**%**	**N**	**CCI*1000**	**95% CI**		**p-value**
	**1490495**	**100**	**47736**	**100**	**1538231**	**31.00**	**30.80**	**31.30**	
**EDUCATIONAL LEVEL**									
**Low**	610746	40.98	19999	41.9	630745	31.71	31.27	32.15	<0.001
**Medium**	538178	36.11	17707	37.09	555885	31.85	31.39	32.33	<0.001
**High**	341571	22.92	10030	21.01	351601	28.53	27.97	29.09	
**SEX**									
**Male**	662069	44.42	22384	46.89	684453	32.70	32.28	33.13	<0.001
**Female**	828426	55.58	25352	53.11	853778	29.69	29.33	30.06	
**AGE CLASSES**									
**35-44**	234567	15.74	8487	17.78	243054	34.92	34.18	35.67	<0.001
**45-54**	364192	24.43	13653	28.6	377845	36.13	35.53	36.74	<0.001
**55-64**	329347	22.1	11053	23.15	340400	32.47	31.87	33.08	<0.001
**65-74**	260956	17.51	6276	13.15	267232	23.48	22.91	24.07	<0.001
**75-79**	112803	7.57	2653	5.56	115456	22.98	22.12	23.87	<0.001
**80+**	188630	12.66	5614	11.76	194244	28.90	28.16	29.67	
				**MARCH-MAY**					
				**SARS-CoV-2 INFECTION**					
	**No**		**Yes**		**All**				
	**N**	**%**	**N**	**%**	**N**	**CCI*1000**	**95% CI**		**p-value**
	**1535446**	**100**	**2191**	**100**	**1537637**	**1.42**	**1.37**	**1.49**	
**EDUCATIONAL LEVEL**	629342	40.99	995	45.41	630337				
**Low**						1.58	1.48	1.68	<0.001
**Medium**	555158	36.16	603	27.52	555761	1.08	1.00	1.18	<0.001
**High**	350946	22.86	593	27.07	351539	1.69	1.56	1.83	
**SEX**									
**Male**	683062	44.49	1089	49.7	684151	1.59	1.50	1.69	<0.001
**Female**	852384	55.51	1102	50.3	853486	1.29	1.22	1.37	
**AGE CLASSES**	242858	15.82	195	8.9	243053				
**35-44**						0.80	0.70	0.92	<0.001
**45-54**	377417	24.58	417	19.03	377834	1.10	1.00	1.21	<0.001
**55-64**	339917	22.14	440	20.08	340357	1.29	1.18	1.42	<0.001
**65-74**	266762	17.37	357	16.29	267119	1.34	1.20	1.48	<0.001
**75-79**	115176	7.5	192	8.76	115368	1.66	1.44	1.92	<0.001
**80+**	193316	12.59	590	26.93	193906	3.04	2.81	3.30	
				**JUNE-AUGUST**					
				**SARS-CoV-2 INFECTION**					
	**No**		**Yes**		**All**				
	**N**	**%**	**N**	**%**	**N**	**CCI*1000**	**95% CI**		**p-value**
	**1536365**	**100**	**849**	**100**	**1537214**	**0.55**	**0.52**	**0.59**	

**EDUCATIONAL LEVEL**									
**Low**	629705	40.99	327	38.52	630032	0.52	0.47	0.58	<0.001
**Medium**	555373	36.15	320	37.69	555693	0.58	0.52	0.64	<0.001
**High**	351287	22.86	202	23.79	351489	0.57	0.50	0.66	
**SEX**									
**Male**	683500	44.49	422	49.71	683922	0.62	0.56	0.68	<0.001
**Female**	852865	55.51	427	50.29	853292	0.50	0.46	0.55	
**AGE CLASSES**									
**35-44**	242885	15.81	166	19.55	243051	0.68	0.59	0.80	<0.001
**45-54**	377567	24.58	260	30.62	377827	0.69	0.61	0.78	<0.001
**55-64**	340152	22.14	182	21.44	340334	0.53	0.46	0.62	<0.001
**65-74**	266956	17.38	96	11.31	267052	0.36	0.29	0.44	<0.001
**75-79**	115265	7.5	47	5.54	115312	0.41	0.31	0.54	<0.001
**80+**	193540	12.6	98	11.54	193638	0.51	0.42	0.62	

SEPTEMBER-DECEMBER

SARS-CoV-2 INFECTION

	**No**		**Yes**		**All**				
	**N**	**%**	**N**	**%**	**N**	**CCI*1000**	**95% CI**		**p-value**
	**1493004**	**100**	**44093**	**100**	**1537097**	**28.69**	**28.42**	**28.96**	
**EDUCATIONAL LEVEL**									
**Low**	611681	40.97	18261	41.41	629942	28.99	28.57	29.41	<0.001
**Medium**	539016	36.1	16659	37.78	555675	29.98	29.53	30.44	<0.001
**High**	342307	22.93	9173	20.8	351480	26.10	25.57	26.64	
**SEX**									
**Male**	663311	44.43	20566	46.64	683877	30.07	29.66	30.49	<0.001
**Female**	829693	55.57	23527	53.36	853220	27.57	27.23	27.93	
**AGE CLASSES**									
**35-44**	234926	15.74	8125	18.43	243051	33.43	32.71	34.16	<0.001
**45-54**	364861	24.44	12965	29.4	377826	34.31	33.73	34.91	<0.001
**55-64**	329945	22.1	10388	23.56	340333	30.52	29.94	31.12	<0.001
**65-74**	261319	17.5	5708	12.95	267027	21.38	20.83	21.94	<0.001
**75-79**	112972	7.57	2325	5.27	115297	20.17	19.36	21.00	<0.001
**80+**	188981	12.66	4582	10.39	193563	23.67	23.00	24.37	

*$\chi 2$ p-value after Bonferroni adjustment for multiple comparison.

Table S2. Univariate and multivariable log-binomial regression model. RR, 95% CI and p-value.

	**ENTIRE PERIOD**							
	**Unajusted analysis**				**Adjusted analysis**			
	**RR**	**95% CI**		**p-value**	**RR**	**95% CI**		**p-value**
**EDUCATIONAL LEVEL**								
**Low**	1.11	1.09	1.14	<.0001	1.23	1.20	1.26	<.0001
**Medium**	1.12	1.09	1.14	<.0001	1.12	1.09	1.15	<.0001
**High**	1.00				1.00			
**SEX**								
**Male**	1.10	1.08	1.11	<.0001	1.09	1.07	1.11	<.0001
**Female**	1.00				1.00			
**AGE CLASSES**								
**35-44**	1.11	1.07	1.14	<.0001	1.28	1.24	1.33	<.0001
**45-54**	1.17	1.14	1.20	<.0001	1.31	1.27	1.35	<.0001
**55-64**	1.07	1.04	1.10	<.0001	1.17	1.13	1.20	<.0001
**65-74**	0.79	0.76	0.82	<.0001	0.83	0.80	0.86	<.0001
**75-79**	0.79	0.76	0.83	<.0001	0.80	0.77	0.84	<.0001
**80+**	1.00				1.00			
	**MARCH-MAY**							
	**Unajusted analysis**				**Adjusted analysis**			
	**RR**	**95% CI**		**p-value**	**RR**	**95% CI**		**p-value**
**EDUCATIONAL LEVEL**								
**Low**	0.94	0.85	1.04	0.2003	0.71	0.64	0.79	<.0001
**Medium**	0.64	0.57	0.72	<.0001	0.64	0.57	0.71	<.0001
**High**	1.00				1.00			
**SEX**								
**Male**	1.21	1.12	1.31	<.0001	1.29	1.19	1.41	<.0001
**Female**	1.00				1.00			
**AGE CLASSES**								
**35-44**	0.27	0.24	0.31	<.0001	0.24	0.21	0.29	<.0001
**45-54**	0.35	0.31	0.39	<.0001	0.34	0.30	0.39	<.0001
**55-64**	0.42	0.38	0.48	<.0001	0.41	0.36	0.46	<.0001
**65-74**	0.44	0.39	0.50	<.0001	0.42	0.37	0.48	<.0001
**75-79**	0.56	0.48	0.65	<.0001	0.54	0.45	0.63	<.0001
**80+**	1.00				1.00			
	**JUNE-AUGUST**							
	**Unajusted analysis**				**Adjusted analysis**			
	**RR**	**95% CI**		**p-value**	**RR**	**95% CI**		**p-value**
**EDUCATIONAL LEVEL**								
**Low**	0.90	0.76	1.08	0.2547	1.02	0.85	1.22	0.8492
**Medium**	1.00	0.84	1.19	0.9821	1.01	0.85	1.21	0.9071
**High**	1.00				1.00			
**SEX**								
**Male**	1.24	1.10	1.40	0.0005	1.22	1.06	1.39	0.0044
**Female**	1.00				1.00			
**AGE CLASSES**								
**35-44**	1.47	1.17	1.85	0.0008	1.33	1.03	1.72	0.0318
**45-54**	1.39	1.11	1.73	0.0035	1.34	1.05	1.70	0.017
**55-64**	1.07	0.84	1.35	0.5865	1.04	0.81	1.34	0.7529

	RR	95% CI		p-value	RR	95% CI		p-value
**65-74**	0.71	0.54	0.93	0.0142	0.70	0.53	0.93	0.0143
**75-79**	0.83	0.59	1.15	0.2609	0.80	0.56	1.13	0.2036
**80+**	1.00				1.00			

**SEPTEMBER-DECEMBER**								
	**Unajusted analysis**				**Adjusted analysis**			
	**RR**	**95% CI**		**p-value**	**RR**	**95% CI**		**p-value**
**EDUCATIONAL LEVEL**								
**Low**	1.11	1.08	1.14	<.0001	1.27	1.23	1.30	<.0001
**Medium**	1.15	1.12	1.18	<.0001	1.15	1.13	1.18	<.0001
**High**	1.00				1.00			
**SEX**								
**Male**	1.08	1.07	1.10	<.0001	1.07	1.05	1.09	<.0001
**Female**	1.00				1.00			
**AGE CLASSES**								
**35-44**	1.28	1.24	1.33	<.0001	1.51	1.46	1.57	<.0001
**45-54**	1.35	1.31	1.39	<.0001	1.53	1.48	1.59	<.0001
**55-64**	1.22	1.18	1.26	<.0001	1.34	1.30	1.39	<.0001
**65-74**	0.87	0.84	0.91	<.0001	0.93	0.89	0.96	0.0002
**75-79**	0.85	0.81	0.89	<.0001	0.86	0.82	0.90	<.0001
**80+**	1.00				1.00			

*Adjusted for age and sex. p-value after Bonferroni adjustment for multiple comparison.

Table S3: Deaths within 30 days of infection by educational level, sex, and age. Crude mortality rate (CMR) with 95% CI and and χ2 p-value. Rome, 35+ years old, 1-03-2020 to 31-12-2020.

	**ENTIRE PERIOD**								
	**Dead within 30 days of infection**								
	**No**		**Yes**		**All**				
	**N**	**%**	**N**	**%**	**N**	**CMR*1000**	**95% CI**		**p-value**
	**45455**	**100**	**2281**	**100**	**47736**	**47.78**	**45.86**	**49.79**	
**EDUCATIONAL LEVEL**									
**Low**	18385	40.45	1614	70.76	19999	80.70	76.86	84.74	<0.001
**Medium**	17269	37.99	438	19.2	17707	24.74	22.53	27.17	<0.001
**High**	9801	21.56	229	10.04	10030	22.83	20.06	25.99	
**SEX**									
**Male**	21100	46.42	1284	56.29	22384	57.36	54.31	60.59	<0.001
**Female**	24355	53.58	997	43.71	25352	39.32	36.96	41.84	
**AGE CLASSES**									
**35-44**	8477	18.65	10	0.44	8487	1.18	0.63	2.19	<0.001
**45-54**	13605	29.93	48	2.1	13653	3.52	2.65	4.67	<0.001
**55-64**	10911	24	142	6.23	11053	12.85	10.90	15.14	<0.001
**65-74**	5865	12.9	411	18.02	6276	65.49	59.45	72.14	<0.001
**75-79**	2308	5.08	345	15.12	2653	130.04	117.02	144.51	<0.001
**80+**	4289	9.44	1325	58.09	5614	236.03	223.64	249.08	
	**MARCH-MAY**								
	**Dead within 30 days of infection**								
	**No**		**Yes**		**All**				
	**N**	**%**	**N**	**%**	**N**	**CMR*1000**	**95% CI**		**p-value**
	**1847**	**100**	**344**	**100**	**2191**	**157.00**	**141.27**	**174.51**	
**EDUCATIONAL LEVEL**									
**Low**	750	40.61	245	71.22	995	246.23	217.25	279.07	<0.001
**Medium**	549	29.72	44	12.79	593	91.21	70.03	118.80	<0.001
**High**	548	29.67	55	15.99	603	74.20	55.22	99.71	
**SEX**									
**Male**	951	51.49	151	43.9	1102	177.23	153.91	204.09	<0.001
**Female**	896	48.51	193	56.1	1089	137.02	116.82	160.72	
**AGE CLASSES**									
**35-44**	193	10.45	2	0.58	195	10.26	2.56	41.01	<0.001
**45-54**	410	22.2	7	2.03	417	16.79	8.00	35.21	<0.001
**55-64**	423	22.9	17	4.94	440	38.63	24.02	62.15	<0.001
**65-74**	305	16.51	52	15.12	357	145.66	110.99	191.15	<0.001
**75-79**	142	7.69	50	14.53	192	260.41	197.37	343.59	<0.001
**80+**	374	20.25	216	62.79	590	366.12	320.40	418.32	
	**JUNE-AUGUST**								
	**Dead within 30 days of infection**								
	**No**		**Yes**		**All**				
	**N**	**%**	**N**	**%**	**N**	**CMR*1000**	**95% CI**		**p-value**
	**814**	**100**	**35**	**100**	**849**	**41.23**	**29.60**	**57.42**	

**EDUCATIONAL LEVEL**									
**Low**	301	36.98	26	74.29	327	79.51	54.14	116.78	<0.001
**Medium**	199	24.45	3	8.57	202	18.75	8.42	41.74	<0.001
**High**	314	38.57	6	17.14	320	14.85	4.79	46.05	
**SEX**					429				
**Male**	406	49.75	23	65.71	429	28.44	16.15	50.07	<0.001
**Female**	410	50.25	12	34.29	422	53.86	35.79	81.06	
**AGE CLASSES**									
**35-44**	166	20.39	.	.	166	.	.	.	<0.001
**45-54**	259	31.82	1	2.86	260	3.85	0.54	27.30	<0.001
**55-64**	182	22.36	.	.	182	.	.	.	<0.001
**65-74**	92	11.3	4	11.43	96	41.66	15.64	111.01	<0.001
**75-79**	42	5.16	5	14.29	47	106.38	44.28	255.59	<0.001
**80+**	73	8.97	25	71.43	98	255.10	172.37	377.53	

SEPTEMBER-DECEMBER

Dead within 30 days of infection

	**No**		**Yes**		**All**				
	**N**	**%**	**N**	**%**	**N**	**CMR*1000**	**95% CI**		**p-value**
	**42431**	**100**	**1662**	**100**	**44093**	**37.69**	**35.92**	**39.55**	
**EDUCATIONAL LEVEL**									
**Low**	17083	40.26	1178	70.88	18261	64.51	60.93	68.30	<0.001
**Medium**	9013	21.24	160	9.63	9173	19.45	17.44	21.69	<0.001
**High**	16335	38.5	324	19.49	16659	17.44	14.94	20.37	
**SEX**									
**Male**	22801	53.74	726	43.68	23527	45.51	42.69	48.52	<0.001
**Female**	19630	46.26	936	56.32	20566	30.86	28.69	33.19	
**AGE CLASSES**									
**35-44**	8117	19.13	8	0.48	8125	0.98	0.49	1.97	<0.001
**45-54**	12927	30.47	38	2.29	12965	2.93	2.13	4.03	<0.001
**55-64**	10279	24.23	109	6.56	10388	10.49	8.70	12.66	<0.001
**65-74**	5403	12.73	305	18.35	5708	53.43	47.76	59.78	<0.001
**75-79**	2081	4.9	244	14.68	2325	104.95	92.57	118.98	<0.001
**80+**	3624	8.54	958	57.64	4582	209.09	196.24	222.75	

*$\chi 2$ p-value after Bonferroni adjustment for multiple comparison.

Table S4: Univariate and multivariable Cox regression model. HR, 95% CI and p-value.

ENTIRE PERIOD								
	**Unajusted analysis**				**Adjusted analysis**			
	**HR**	**95% CI**		**p-value**	**HR**	**95% CI**		**p-value**
**EDUCATIONAL LEVEL**								
**Low**	3.64	3.17	4.18	<.0001	1.37	1.19	1.58	<.0001
**Medium**	1.08	0.92	1.27	0.3292	1.14	0.97	1.33	0.1197
**High**	1.00				1.00			
**SEX**								
**Male**	1.47	1.35	1.60	<.0001	1.95	1.79	2.12	<.0001
**Female**	1.00				1.00			
**AGE CLASSES**								
**35-44**	0.004	0.002	0.008	<.0001	0.01	0.00	0.01	<.0001
**45-54**	0.013	0.010	0.017	<.0001	0.01	0.01	0.02	<.0001
**55-64**	0.048	0.040	0.057	<.0001	0.05	0.04	0.06	<.0001
**65-74**	0.250	0.223	0.279	<.0001	0.23	0.21	0.26	<.0001
**75-79**	0.512	0.455	0.576	<.0001	0.48	0.42	0.54	<.0001
**80+**	1.00				1.00			

MARCH-MAY								
	**Unajusted analysis**				**Adjusted analysis**			
	**HR**	**95% CI**		**p-value**	**HR**	**95% CI**		**p-value**
**EDUCATIONAL LEVEL**								
**Low**	3.65	2.65	5.03	<.0001	1.39	0.99	1.96	0.0562
**Medium**	1.23	0.83	1.83	0.3015	1.11	0.75	1.66	0.6034
**High**	1.00				1.00			
**SEX**								
**Male**	1.32	1.06	1.63	0.0117	1.80	1.45	2.24	<.0001
**Female**	1.00				1.00			
**AGE CLASSES**								
**35-44**	0.02	0.01	0.09	<.0001	0.03	0.01	0.11	<.0001
**45-54**	0.04	0.02	0.08	<.0001	0.04	0.02	0.09	<.0001
**55-64**	0.09	0.05	0.14	<.0001	0.09	0.05	0.14	<.0001
**65-74**	0.34	0.25	0.46	<.0001	0.33	0.24	0.45	<.0001
**75-79**	0.65	0.48	0.89	0.0066	0.64	0.47	0.87	0.0043
**80+**	1.00				1.00			

JUNE-AUGUST								
	**Unajusted analysis**				**Adjusted analysis**			
	**HR**	**95% CI**		**p-value**	**HR**	**95% CI**		**p-value**
**EDUCATIONAL LEVEL**								
**Low**	5.52	1.67	18.22	0.0051	0.91	0.26	3.12	0.8748
**Medium**	1.27	0.32	5.07	0.7378	1.07	0.27	4.34	0.9211
**High**	1.00				1.00			
**SEX**								
**Male**	0.52	0.26	1.05	0.0694	0.56	0.81	0.40	0.3396
**Female**	1.00				1.00			
**AGE CLASSES**								
**35-44**								

	HR	95% CI		p-value	HR	95% CI		p-value
**45-54**	0.013	0.002	0.099	<.0001	0.013	0.002	0.100	<.0001
**55-64**								
**65-74**	0.149	0.052	0.427	0.0004	0.151	0.052	0.440	0.0005
**75-79**	0.390	0.149	1.019	0.0546	0.395	0.149	1.044	0.061
**80+**	1.00				1.00			

SEPTEMBER-DECEMBER

	**Unajusted analysis**				**Adjusted analysis**			
	**HR**	**95% CI**		**p-value**	**HR**	**95% CI**		**p-value**
**EDUCATIONAL LEVEL**								
**Low**	3.79	3.21	4.47	<.0001	1.43	1.21	1.69	<.0001
**Medium**	1.12	0.92	1.35	0.2566	1.18	0.97	1.42	0.0942
**High**	1.00				1.00			
**SEX**								
**Male**	1.49	1.35	1.64	<.0001	1.98	1.79	2.18	<.0001
**Female**	1.00				1.00			
**AGE CLASSES**								
**35-44**	0.004	0.002	0.008	<.0001	0.004	0.002	0.009	<.0001
**45-54**	0.012	0.009	0.017	<.0001	0.013	0.009	0.018	<.0001
**55-64**	0.045	0.037	0.055	<.0001	0.044	0.036	0.053	<.0001
**65-74**	0.233	0.205	0.265	<.0001	0.217	0.191	0.248	<.0001
**75-79**	0.469	0.407	0.539	<.0001	0.435	0.378	0.501	<.0001
**80+**	1.00				1.00			

*Adjusted for age and sex. p-value after Bonferroni adjustment for multiple comparison.
